# 1134. The Effect of Telehealth Antimicrobial Stewardship Program on Antimicrobial Use in a Pediatric Intensive Care Unit

**DOI:** 10.1093/ofid/ofab466.1327

**Published:** 2021-12-04

**Authors:** Mohammad Alghounaim, Ahmed Abdelmoniem, Mohamed Elseadawy, Mohammad Surour, Mohamed Basuni, Jesse Papenburg, Abdulla Alfraij

**Affiliations:** 1 Amiri Hospital, Kuwait City, Al Asimah, Kuwait; 2 Farwaniyah Hospital, Sabah Alsalem, Al Farwaniyah, Kuwait; 3 Departments of Pediatrics and Medical Microbiology, McGill University Health Centre, Montreal, QC, Canada, Montreal, Quebec, Canada

## Abstract

**Background:**

Inappropriate antimicrobial use is common in pediatric intensive care units (PICU). We aimed to evaluate the effect of telehealth antimicrobial stewardship program (ASP) on the rate of PICU antimicrobial use in a center without a local infectious diseases consultation service.

**Methods:**

Aretrospective cohort study was performed between October 1^st^, 2018 and October 31^st^, 2020 in Farwaniyah Hospital PICU, a 20-bed unit. All pediatric patients who were admitted to PICU and received systemic antimicrobials during the study period were included and followed until hospital discharge. Patients admitted to the PICU prior to the study period but still receiving intensive care during the study period were excluded. Weekly prospective audit and feedback on antimicrobial use was provided starting October 8^th^, 2019 (post-ASP period) by the ASP team. A pediatric infectious diseases specialist would join ASP rounds remotely. Descriptive analyses and a pre-post intervention comparison of days of therapy (DOT) were used to assess the effectiveness of the ASP intervention

**Results:**

There were 272 and 152 PICU admissions before and after initiation of ASP, respectively. Bronchiolitis and pneumonia were the most common admission diagnoses, together compromising 60.7% and 61.2% pre- and post-ASP. Requirement for respiratory support was higher post-ASP (76.5% vs 91.5%, p< 0.001). Average monthly antimicrobial use decreased from 92.2 (95% CI 74.5 to 100) to 48.5 DOT/1,000 patient-days (95% CI 24.6 to 72.2, P < 0.05) (figure). A decline in DOT was observed across all antibiotic classes, except for ceftriaxone and clarithromycin. No effect on length of PICU stay, hospital length of stay, or mortality was observed. Most (89.7%) ASP recommendations were followed fully or partially changes in antimicrobial days of therapy (DOT)/1,000 patient-days over time. The dashed line represents the start of the antimicrobial stewardship program (ASP)

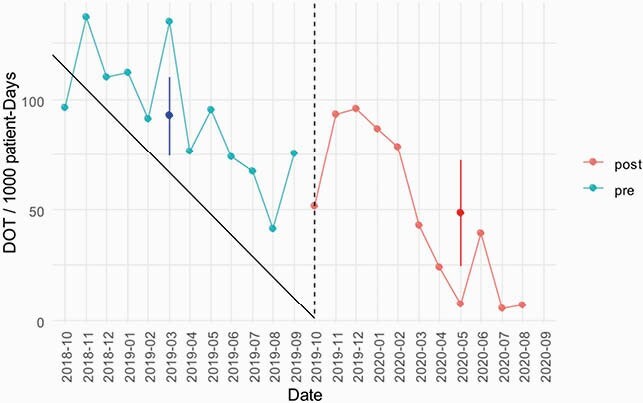

**Conclusion:**

In settings where infectious diseases services are not available, telehealth stewardship can be effectively implemented and associated with a significant reduction of antimicrobial use.

**Disclosures:**

**Jesse Papenburg, MD**, **AbbVie** (Grant/Research Support, Other Financial or Material Support, Personal fees)**Medimmune** (Grant/Research Support)**Sanofi Pasteur** (Grant/Research Support)**Seegene** (Grant/Research Support, Other Financial or Material Support, Personal fees)

